# Early Risk Prediction for Biologic Therapy in Psoriasis Using Machine Learning Models Based on Routine Health Records

**DOI:** 10.3390/jcm14186421

**Published:** 2025-09-11

**Authors:** Tair Lax, Noga Fallach, Edia Stemmer, Guy Shrem, Mali Salmon-Divon

**Affiliations:** 1 Department of Molecular Biology, Ariel University, Ariel 4070000, Israel; tairl@ariel.ac.il (T.L.);; 2 Fertility Clinic, Clalit Health Services, Migdal HaEmek 2303001, Israel; shgaish@clalit.org.il; 3 Adelson School of Medicine, Ariel University, Ariel 4070000, Israel

**Keywords:** skin diseases, retrospective studies, machine learning, biological products

## Abstract

**Background**: Psoriasis is a chronic inflammatory skin disease with a variable course. Early identification of patients likely to require biologic therapy may help reduce complications and optimize care. In this study, we developed machine learning (ML) models to predict future biologic therapy use in psoriasis patients. **Methods**: We conducted a retrospective study using electronic health records (EHR) from Clalit Health Services in Israel, including psoriasis patients who started biologic therapy and matched psoriasis controls. Predictors included demographics, comorbidities, treatment history, and laboratory test results. KNN, SVM, Random Forest, and Logistic Regression ML models were trained on data from either the first five years post-onset or the five years preceding biologic therapy. Performance was evaluated on a held-out test set using AUC-ROC, precision, recall, and F1-score, with an emphasis on recall to maximize identification of true positive cases. **Results**: The best-performing models incorporated clinical, demographic, and laboratory data. Using data from the first five years after onset, the SVM model achieved the highest performance (AUC = 0.83, recall = 0.7). For data from the five years preceding biologic therapy, the Random Forest model performed best (AUC = 0.93, recall = 0.95). Key predictors included comorbid immune-mediated conditions, topical treatment frequency, and markers of inflammation and metabolism. **Conclusions**: EHR-based ML models, particularly those incorporating routine laboratory, demographic, and clinical data, can effectively predict future biologic therapy use in psoriasis patients. Model performance may be improved with larger cohorts and more complete clinical and laboratory data.

## 1. Introduction

Psoriasis is a chronic immune-mediated inflammatory disease (IMID) characterized by skin lesions resulting from abnormal epidermal growth and differentiation. The disease develops when the immune system’s innate and adaptive components interact abnormally with skin cells, leading to inflammation and the formation of psoriatic lesions [1].

Psoriasis treatment varies depending on its severity. Mild cases are generally managed with topical treatments such as corticosteroids and vitamin D analogs. Moderate to severe psoriasis may require phototherapy or systemic medications, including methotrexate and cyclosporine. Severe cases often necessitate biological therapies targeting specific immune pathways, such as TNF-alpha or IL-17 inhibitors [2]. Regardless of severity, lifestyle modifications are essential for effective disease management [3].

Finding the best treatment for psoriasis is often a lengthy and challenging process due to the disease’s variability and suboptimal responses to therapies [4]. Patients undergo trial and error with different treatments, ranging from topical options to systemic medications, which can be influenced by factors like disease severity and side effects. Moreover, the high cost of newer treatments and limited access to specialized care further complicate management. Developing predictive tools to identify patients at risk of requiring advanced treatments such as biologic therapy could improve outcomes by enabling earlier interventions, reducing complications, optimizing care, and ultimately lowering healthcare costs.

While several studies have used machine learning (ML) to predict treatment outcomes of biologic therapy in psoriasis [5,6,7], we found no studies specifically predicting the risk of initiating biologic therapy in psoriasis. Two studies have addressed this question in inflammatory bowel disease (IBD): one developed an ML model using clinical and laboratory data to predict 5-year risk of biologic initiation, achieving an AUC of 0.81 in external validation [8], and another predicted the probability of biologic therapy based on pre-treatment and demographic variables, reporting an accuracy of 0.74 [9].

In this study, we aimed to evaluate ML-based methods for predicting the need for biologic therapy in psoriasis patients using data derived from electronic health records (EHR) from Clalit Health Services (CHS) in Israel. The analysis used baseline characteristics, including gender, age at disease onset, prior treatments, co-existing IMID conditions, and laboratory test measurements.

## 2. Materials and Methods

### 2.1. Data Source

The study is based on electronic health records extracted from the CHS database, the largest Health Maintenance Organization (HMO) in Israel, serving a population of more than 4.5 million people as of 2024. Data was extracted by the North District’s Research Data Center, using the Clalit Research Data sharing platform for de-identified data powered by MDClone (https://www.mdclone.com, accessed on 12 May 2022). The electronic health records were recorded between 1998 and 2022 (excluding some retroactive diagnoses from before 1998) and contained clinical and administrative data collected in hospitals (inpatient clinics and emergency room settings), primary care clinics, pharmacies, laboratories, and diagnostic and imaging centers. The data are also linked to national databases providing socio-demographic information related to patients and clinics. The data were specifically extracted from the CHS database focusing on inflammatory diseases.

### 2.2. Inclusion Criteria

Patients were included in the psoriasis group if they had at least one documented diagnosis of psoriasis vulgaris, pustular psoriasis, parapsoriasis, psoriasis inverse, or psoriatic arthritis, identified by ICD-10 codes L40, L40.1, L41, L44.8, L40.54, L40.59, or ICD-9 codes 696, 696.3, 696.2, 696.1, 696.8, and 696.0. Inclusion also required at least one recorded purchase of a topical medication relevant to psoriasis, identified by ATC codes starting with D05A, D07A, D07X, D11AH01, or D11AH02. The date of psoriasis onset was determined as the earlier date between the first recorded psoriasis diagnosis and the first topical medication purchase. To ensure data quality, we excluded patients whose onset date coincided with their first entry in the database, as this likely indicated retrospective data. Additionally, patients with records of biologic medication use prior to the defined onset date were excluded.

### 2.3. Cohort Balancing and Data Partitioning

To balance the cohort, each patient receiving biologic therapy was matched with two other patients who did not receive biologic therapy. Matching criteria included gender, age at psoriasis onset, and the duration of follow-up before and after onset. This process was performed using the “MatchIt” package (version 4.5.4) in R (version 4.2.3). We defined a data extraction date, i.e., index date, for each patient in the Biologic Therapy Group as the biologic treatment initiation date. To ensure that data were comparable between the two groups prior to biologic treatment, we defined the index date for the Conventional Therapy group as their matched biologic-treated patients’ index date. This allowed for a consistent comparison of data up to the point of biologic therapy initiation, ensuring that both groups had data extracted at the same relative time.

To evaluate the ML models, we used a 90/10 train/test split of the selected patients. The 10% test set was not included in the model development process and was used exclusively as hold-out data for final model evaluation. A graphical representation of the cohort selection process is provided in Supplementary Figure S1.

### 2.4. Biologic Medications

Biologic therapy initiation date was defined as the first purchase of the biologic medications Remicade, Enbrel, Humira, Stelara, Cosentyx, Taltz, Tremfya, Ilumya, Skyrizi, and Cimzia. No records for Bimzelx or Siliq were available in the database. We retrieved medication records using the corresponding ATC-5 codes (L04AB01, L04AB02, L04AB04, L04AB05, L04AC05, L04AC10, L04AC13, L04AC16, L04AC17, L04AC18) to ensure inclusion of biosimilar products.

### 2.5. Predictor Variables

To predict which patients were more likely to receive biologic therapy, we collected data from disease onset up to five years post-onset or until the index date, whichever came first. Additionally, we performed further analysis using data from the five years preceding the index date. For each laboratory test, we calculated several statistical measures: count, mean, standard deviation, interquartile range (IQR), range (the interval between the maximum and minimum test values), minimum, and maximum test values per patient. Additional variables included gender and age at disease onset. Treatment history was assessed based on the number of topical treatment purchases within the mentioned periods, categorized by ATC codes (D05A, D07A + D07X, and D11AH01 + D11AH02). Comorbidities documented up to five years after psoriasis onset or before the index date, included other IMIDs, such as psoriatic arthritis (PsA), atopic dermatitis (AD), IBD, ankylosing spondylitis (AS), multiple sclerosis (MS), and rheumatoid arthritis (RA). Socioeconomic status was classified as high, medium, or low.

### 2.6. Model Development

To predict biologic therapy, we developed multiple ML models to determine the best-performing one. The models evaluated in this study included k-Nearest Neighbors (KNN), Support Vector Machine (SVM), Random Forest (RF), and Logistic Regression (LR). All model training, hyperparameter tuning, and evaluation were performed using the *scikit-learn* package (version 1.2.1) in Python (version 3.9.7).

#### 2.6.1. Feature Engineering and Selection

We calculated the count, mean, standard deviation, interquartile range (IQR), range, minimum, and maximum laboratory test values per patient. However, a combination of patient laboratory tests’ mean and range yielded the best results for prediction. Adding other laboratory measurement features introduced multicollinearity and did not significantly improve the model’s performance. In addition, feature selection methods did not enhance the results and were not applied. To reduce multicollinearity among the included features, we assessed pairwise correlations between variables (Supplementary Figures S2 and S3). Features with high intercorrelation (Pearson’s |r| > 0.8) were considered redundant. For each highly correlated pair, one feature was excluded from the analysis (Supplementary Tables S1 and S2).

We ran the models twice: the first time based on laboratory data alone and the second time combined with non-laboratory data. Non-laboratory data included age of onset, presence of other IMIDs, counts of topical treatment purchases, socioeconomic status, and gender.

#### 2.6.2. Data Imputation and Scaling

Patients who had more than 12 lab features with missing data were excluded from the analysis. In addition, predictors with more than 20% missing data (including information about BMI, smoking status, physical activity status, blood pressure measurements, and specific lab tests) were excluded. For the remaining missing values, we applied iterative imputation, performed separately on train and test data, with a maximum of 100 iterations using the IterativeImputer function from the scikit-learn Python package (version 1.2.1). All numeric features were standardized using the StandardScaler function from the same package to ensure a consistent distribution across variables.

#### 2.6.3. Training and Validation Approach

Model performance during training was assessed using five-fold cross-validation. The training dataset was split into five subsets, where each fold served as a validation set once, while the remaining four folds were used for training. We used the StratifiedKFold function to ensure that each set contains approximately the same percentage of samples of each target class as the complete set.

#### 2.6.4. Hyperparameter Tuning

Hyperparameter tuning was conducted using the GridSearchCV package with cross-validation to optimize the models’ performance. The ROC_AUC metric was applied to balance overall performance and was selected because it also provided strong recall, our primary priority given its clinical relevance.

#### 2.6.5. Class Imbalance Handling

Given the imbalance in the dataset, where a smaller proportion of patients received biologic therapy, we performed undersampling of the conventional therapy group at a ratio of 1:2 to balance the classes. Without this adjustment, the models exhibited bias toward the conventional Therapy group. Additionally, the ‘class_weight’ parameter was applied for SVM, LR, and RF to further mitigate bias toward the majority class.

#### 2.6.6. Model Performance Evaluation

After identifying the best models during training, we evaluated their performance on the 10% hold-out test dataset, which was not involved in the training process. Model performance was assessed using the area under the receiver operating characteristic curve (AUC-ROC) to evaluate classification performance, along with F1-score, precision, and recall to comprehensively assess model effectiveness.

#### 2.6.7. Features Importance

We evaluated feature importance by calculating the permutation importance of the trained models on the hold-out test data. This method assesses the importance of each feature by measuring the decrease in model performance when the feature’s values are randomly shuffled. A greater drop in performance indicates a more influential feature. The resulting permutation importance scores were used to identify the most impactful predictors of biologic therapy use.

### 2.7. Statistical Analysis

The time from psoriasis onset to initiation of biologic treatment was estimated using Kaplan–Meier survival curves to account for the varying follow-up times, with patients censored if they did not receive treatment during the observation period. (Time was calculated from psoriasis onset to the date of the last recorded entry in the database, indicating the end of record tracking for the patient.) Only patients whose onset occurred after 1 January 2000, were included in the analysis. The survival analysis was performed using the “ggsurvfit” package (version 0.3.1) in R (version 4.2.3).

### 2.8. Use of Generative AI Tools

During the preparation of this manuscript, the authors used OpenAI’s ChatGPT-3 and ChatGPT-4 to assist with language editing, phrasing, and refining the clarity of ideas.

## 3. Results

### 3.1. Baseline Characteristics

**Figure 1:**
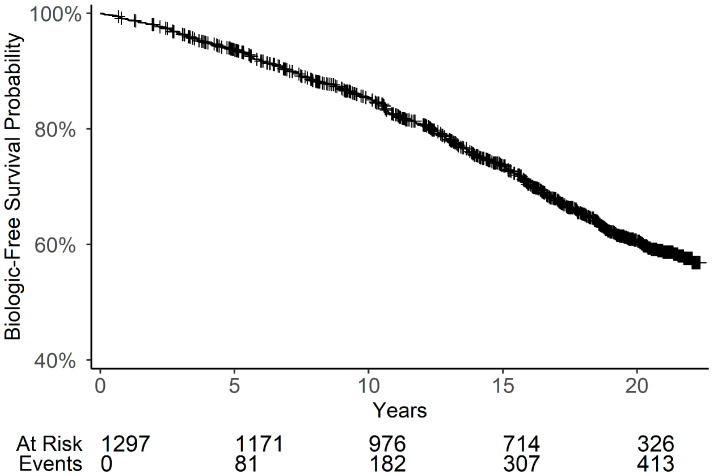
Kaplan–Meier Survival Analysis of Time from Psoriasis Onset to the Initiation of Biologic Therapy. Only patients whose onset occurred after 1 January 2000, were included in the analysis. Y-axis scale is (0.4, 1).

A flow chart of the cohort selection according to the inclusion criteria is depicted in Supplementary Figure S1. A total of 1320 patients met the inclusion criteria, of whom 440 were treated with biologic therapy. A total of 1191 patients (90%) were included in the training data. Among these, 750 patients (63%) were male and 441 (37%) were female. A Kaplan–Meier survival analysis for time to biologic therapy initiation for the entire cohort is shown in Figure 1. The probability of remaining biologic-free gradually declined over time, with only a small proportion of patients receiving biologic therapy within the first five years after disease onset.

### 3.2. Prediction of Biology Therapy Based on Data Recorded in the First 5 Years After Onset

We evaluated the ability of ML models to predict the initiation of biologic therapy in psoriasis patients using data recorded within the first five years after disease onset or until the index date, whichever came first. Two sets of predictive features were used: (A) laboratory test data alone and (B) laboratory test data combined with additional clinical and demographic features. A full list of laboratory tests used in this analysis is provided in Supplementary Table S3.

#### 3.2.1. Model Performance Based on Laboratory Data Alone

**Figure 2:**
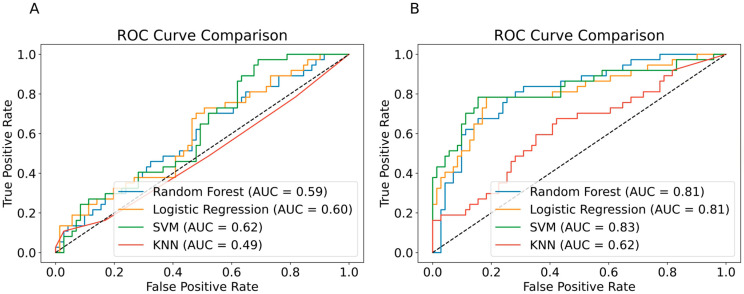
ROC Curves for the Models’ Predictions Based on Laboratory Test Data Alone (**A**) and Laboratory Test Data Combined with Additional Clinical and Demographic Features (**B**). The data used for those predictions were recorded in the first 5 years after onset, or until the index date, whichever came first. The dotted line represents the performance of a random classifier.

Using only laboratory test data resulted in poor predictive performance across all models. While SVM achieved the highest area under the receiver operating characteristic curve (AUC = 0.62), this indicates a discriminative ability barely superior to a random guess. (Figure 2A). The classification metrics supported this finding, with Logistic Regression achieving the highest recall of 0.49 (Table 1, Left).

#### 3.2.2. Model Performance with Additional Clinical and Demographic Features

The inclusion of additional clinical and demographic features significantly improved prediction performance across all models. SVM demonstrated a good overall performance, achieving an AUC of 0.83 (Figure 2B) and best recall scoring of 0.7 (Table 1, Right), highlighting the advantage of incorporating demographic and clinical factors. Retraining models with only clinical and demographic features (excluding laboratory data) slightly improved results for KNN, but led to decreased AUC and recall for both SVM and Logistic Regression.

### 3.3. Prediction of Biologic Therapy Based on Data Recorded 5 Years Before the Index Date

We repeated the analysis using data from the five years preceding the index date. Again, two sets of predictive features were used: (A) laboratory test data alone and (B) laboratory test data combined with additional clinical and demographic features. A full list of laboratory tests used in this analysis is provided in Supplementary Table S4.

#### 3.3.1. Model Performance Based on Laboratory Data Alone

**Figure 3:**
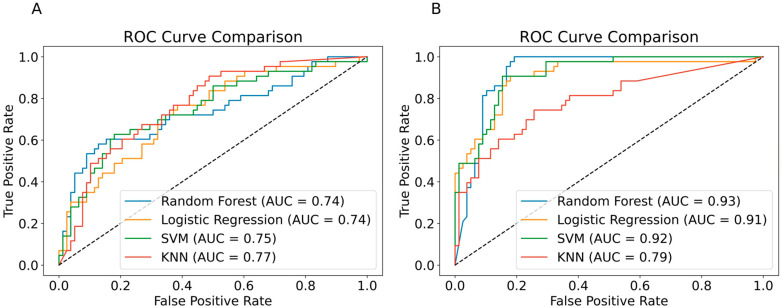
ROC Curves for the Models’ Predictions Based on Laboratory Test Data Alone (**A**) and Laboratory Test Data Combined with Additional Clinical and Demographic Features. (**B**) The data used for those predictions was recorded in the interval of 5 years before the index date. The dotted line represents the performance of a random classifier.

Using only laboratory test data, Logistic Regression showed satisfactory predictive ability, achieving an AUC of 0.74 and a recall of 0.7 (Figure 3A and Table 2, left).

#### 3.3.2. Model Performance with Additional Clinical and Demographic Features

The inclusion of additional clinical and demographic features significantly improved prediction performance across all models. In this analysis, Random Forest demonstrated the best overall performance, achieving an AUC of 0.93 (Figure 3B) and a recall of 0.95 (Table 2, right). Retraining with only clinical and demographic features (excluding laboratory data) yielded similarly strong results across all models, suggesting that clinical information has a notably strong predictive effect in the five years preceding the index date.

### 3.4. Features Importance Analysis

**Figure 4:**
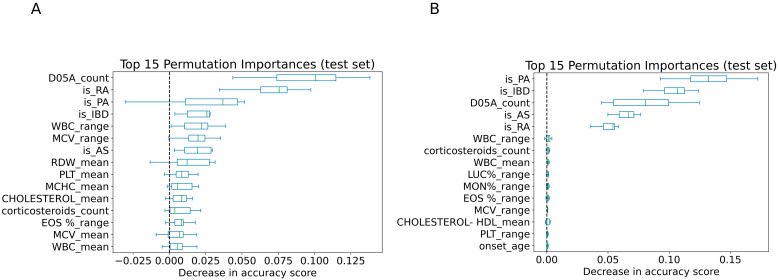
Top 15 Predictors of Biologic Therapy Use. Permutation importance plots displaying the top 15 predictors identified by two models: (**A**) an SVM trained on laboratory tests combined with clinical and demographic data collected within the first 5 years after disease onset, and (**B**) a Random Forest trained on laboratory, clinical, and demographic data from the 5 years preceding the index date. Variables is_PA, is_IBD, is_RA and is_AS indicate the presence of PsA, IBD, RA, and AS, respectively. D05A_count reflects the number of purchases of topical anti-psoriatic medications (ATC code starts with D05A), and corticosteroids_count represents the number of topical corticosteroid purchases (ATC codes start with D07A or D07X).

We applied permutation importance to the best-performing classifiers trained with both laboratory and additional data (SVM and Random Forest), using a held-out test set to identify the most influential predictors of biologic therapy use. The top 15 features with the highest impact on model performance were visualized according to the decrease in accuracy when each feature was permuted. (Figure 4). In both models, an SVM trained on data from the first five years after psoriasis onset (Figure 4A) and a Random Forest trained on data from the five years preceding the index date (Figure 4B). Comorbid autoimmune conditions, including PsA, IBD, RA, AS, contributed substantially to model performance, as did the number of topical anti-psoriatic (D05A count) and corticosteroid purchases. Both models highlighted the importance of various hematological parameters, some of which are known to be associated with the immune system and systemic inflammation. These included white blood cell (WBC) count (WBC mean and range), mean corpuscular volume (MCV mean and range), platelets (PLT mean and range), mean corpuscular hemoglobin concentration (MCHC mean), red cell distribution width (RDW mean), large unstained cells percentage (LUC% range), monocyte percentage (MON% range), and eosinophil percentage (EOS% range). Additionally, metabolic markers such as total cholesterol (CHOLESTEROL mean) and HDL cholesterol (CHOLESTEROL-HDL mean) were also among the top predictors. Age at disease onset was found to be informative in the Random Forest model.

## 4. Discussion

In this study, we developed ML models to predict the need for biologic therapy in psoriasis patients. Models using only laboratory data from the first five years after onset showed limited performance (best AUC 0.62, recall 0.49). Adding clinical and demographic features improved results, with SVM achieving an AUC of 0.83 and a recall of 0.70. Logistic Regression based on laboratory data from the five years before the index date achieved an AUC of 0.74 and a recall of 0.70. Performance further improved with the addition of clinical and demographic features, with Random Forest reaching an AUC of 0.93 and a recall of 0.95. Comorbid autoimmune conditions, the number of topical medication purchases, and hematological and metabolic parameters were the most influential predictors.

Although ML and deep learning techniques have been increasingly applied in psoriasis research [5,6,7,10,11,12], to the best of our knowledge, this is the first study to specifically leverage ML for predicting the future need for biologic treatment in psoriasis patients. Our approach aimed to identify patients at risk of requiring biologic therapy using EHR collected during the early stages of psoriasis. This strategy was driven by the clinical importance of early risk stratification and the opportunity to guide timely intervention based on information already available through routine care. For example, in psoriatic arthritis (PsA), a joint-related complication that affects nearly one-third of individuals with psoriasis, early treatment is crucial for preventing complications and improving quality of life [13]. Recent studies suggest that PsA may even be preventable through the early initiation of biologic therapy [14,15,16,17].

Routine laboratory tests are inexpensive, widely available, and commonly performed in everyday medical practice. Leveraging this advantage, we aimed to develop the models based on this accessible information. The relevance and association of many laboratory tests with psoriasis also motivated our study. For example, components of a complete blood count, such as platelet count and mean platelet volume (MPV), have been identified as potential hematological markers for the presence and severity of psoriasis [18,19,20]. Vitamin D and serum Calcium levels are also strongly linked to the mechanism of psoriasis [21], showing different levels compared to controls [22,23,24], and a moderate association with psoriasis severity [23,25].

Similar to a previous study that distinguished psoriasis patients from healthy controls using routine laboratory tests [10], we aimed to identify patients at risk of severe disease using laboratory data collected within the first five years after disease onset. Our initial model, which relied solely on laboratory features, showed limited predictive performance. This may reflect the difficulty of predicting disease progression in the early stages but could also be due to substantial missing laboratory data within the first five years after onset, which led to the exclusion of many relevant tests. Therefore, we wanted to check how the tests’ timing influences the results. Applying the models using laboratory data recorded during the five years preceding the index date significantly improved recall, accuracy, and AUC. This improvement may reflect both the increased volume of laboratory data available closer to treatment initiation and the presence of clinical deterioration that is captured in those laboratory values.

Model training and evaluation were conducted with an emphasis on recall (sensitivity), prioritizing the correct identification of patients likely to require biologic therapy (true positives), even at the expense of an increased false-positive rate. This approach helps ensure that high-risk patients are not missed, supporting timely and appropriate treatment [26]. The inclusion of additional clinical and demographic features significantly improved recall and AUC in the two analyses.

Psoriasis comorbidities ranked among the top features, reflecting their association with more severe disease that may require biologic therapy. However, it is important to note that in some cases, the biologic medications may have been prescribed primarily for the comorbid condition, with psoriasis being treated only secondarily. Moreover, there are additional comorbidities that may require biologic treatment but were not included in our analysis. The number of topical treatment purchases was also significant and can be easily explained by higher disease severity. Among the most important predictors were also hematological inflammation-related biomarkers like RDW and WBC means, which were evaluated in patients receiving biologic therapy (Supplementary Tables S3 and S4). This finding aligns with previous studies showing that RDW and WBC levels are often elevated in psoriasis patients compared to controls and are positively correlated with disease severity [27,28,29,30]. Regarding lipid-related biomarkers, the mean High-Density Lipoprotein Cholesterol (HDL-C) level was lower in the group receiving biologic therapy (Supplementary Table S4). HDL-C levels are typically reduced in individuals with psoriasis, and impaired HDL function has been associated with increased disease severity [31,32,33]. Other laboratory tests identified as significant predictors were also found to be important in a previous study that used Random Forest models to predict psoriasis [10]. Age at onset was another key feature in the Random Forest model, consistent with our previous work showing that disease onset timing impacts treatment patterns [34].

In summary, we present a proof-of-concept demonstrating the potential of ML to support treatment decision-making in psoriasis. Periodic application of ML models could help clinicians identify patients more likely to require biologic therapy. This approach may help guide personalized treatment strategies and optimize patient management.

Our study has several limitations. First, the relatively low number of patients who received biologic therapy may limit the statistical power and generalizability of the predictive models. Due to the limited data, we tested the models on only 10% of the dataset, which may not fully represent the population. Second, a high proportion of missing values prevented the inclusion of many clinically relevant laboratory tests, such as vitamin D and calcium levels, which may play a role in psoriasis pathophysiology and treatment response. Additionally, important lifestyle and clinical variables, such as smoking status and body mass index (BMI), were excluded due to substantial missingness. These factors are known to influence psoriasis severity and treatment outcomes and should be incorporated in future studies with more complete datasets. Finally, we did not have access to standardized severity measures, such as the Psoriasis Area and Severity Index (PASI), Body Surface Area (BSA), and Dermatology Life Quality Index (DLQI), which limited our ability to directly evaluate disease severity and its influence on treatment decisions.

## 5. Conclusions

This study demonstrates the potential of ML models based on routine laboratory, demographic, and clinical data to predict the future need for biologic therapy in psoriasis. Periodic use of such tools may help support clinical decision-making. Nonetheless, limitations such as missing values and the absence of key variables underscore the need for further training and validation in larger, more comprehensive datasets.

## Figures and Tables

**Table 1:** Metrics Scoring for the Models’ Predictions Based on Data Recorded in the First 5 Years After Disease Onset.

Model	Laboratory Data				Laboratory and Additional Data			
	Precision	Recall	F1 Score	Accuracy	Precision	Recall	F1 Score	Accuracy
Random Forest	0.42	0.46	0.44	0.6	0.7	0.62	0.66	0.78
Logistic Regression	0.38	0.49	0.43	0.56	0.67	0.65	0.66	0.77
SVM	0.36	0.46	0.4	0.54	0.72	0.7	0.71	0.81
KNN	0.33	0.16	0.22	0.6	0.54	0.19	0.28	0.67

**Table 2:** Metrics Scoring for the Models’ Predictions Based on Data Recorded in the 5 Years Before the Index Date.

Model	Laboratory Data				Laboratory and Additional Data			
	Precision	Recall	F1 Score	Accuracy	Precision	Recall	F1 Score	Accuracy
Random Forest	0.53	0.65	0.58	0.67	0.75	0.95	0.84	0.87
Logistic Regression	0.54	0.7	0.61	0.68	0.75	0.84	0.79	0.84
SVM	0.56	0.67	0.61	0.69	0.76	0.88	0.82	0.86
KNN	0.6	0.56	0.58	0.71	0.75	0.42	0.54	0.74

## Data Availability

The data used in this study were accessed under a specific data-sharing agreement with Clalit Health Services (CHS), Israel. Access to these data is restricted to protect patient confidentiality and is available only to researchers who obtain permission from CHS following submission and approval of a detailed research protocol. Data analyses were conducted within the CHS research room as required by CHS policy. Researchers interested in accessing the data or computing code should contact Clalit Health Services directly to inquire about the data access procedure.

## Supplementary Materials

The following supporting information can be downloaded at: https://www.mdpi.com/article/10.3390/jcm14186421/s1, Figure S1: Graphical Representation of the Cohort Selection Process; Figure S2: Correlation Matrix of Laboratory, Demographic, and Clinical Features from the First 5 Years After Psoriasis Onset; Figure S3: Correlation Matrix of Laboratory, Demographic, and Clinical Features from the 5-Year Prior to the Index Date; Table S1: Features with High Intercorrelation (Pearson’s |r| > 0.8) Among Training Data Recorded in the First 5 Years after Onset; Table S2: Features with High Intercorrelation (Pearson’s |r| > 0.8) Among Training Data Recorded in the Interval of 5 Years Before the Index Date; Table S3: Laboratory Tests Used to Build the Models in the First Stage; Table S4: Laboratory Tests Used to Build the Models in the Second Stage.

## Abbreviations

The following abbreviations are used in this manuscript:

IMID	Immune-Mediated Inflammatory Disease
ML	Machine Learning
IBD	Inflammatory Bowel Disease
EHR	Electronic Health Records
CHS	Clalit Health Services
HMO	Health Maintenance Organization
KNN	K-Nearest Neighbors
SVM	Support Vector Machine
RF	Random Forest
LR	Logistic Regression
ROC	Receiver Operating Characteristic
AUC	Area Under Curve
AS	Ankylosing Spondylitis
MS	Multiple Sclerosis
RA	Rheumatoid Arthritis
WBC	White Blood Cell
MPV	Mean Platelet Volume
MCHC	Mean Corpuscular Hemoglobin Concentration
RDW	Red Cell Distribution Width
NEUT%	Neutrophil Percentage
MON%	Monocyte Percentage
LYMP.abs	Lymphocyte Absolute Count
EOS%	Eosinophil Percentage
PsA	Psoriatic Arthritis
HDL-C	High-Density Lipoprotein Cholesterol
BMI	Body Mass Index
PASI	Psoriasis Area and Severity Index
BSA	Body Surface Area
DLQI	Dermatology Life Quality Index
