# Supplementary material for: Early Risk Prediction for Biologic Therapy in Psoriasis Using Machine Learning Models Based on Routine Health Records
Source: J Clin Med. 2025 Sep 11;14(18):6421. doi: 10.3390/jcm14186421 (PMC12470762; doi:10.3390/jcm14186421)
Supplement: Supplementary file 1 [file jcm-14-06421-s001.zip › jcm-3789727-supplementary.pdf]

Supplementary Materials

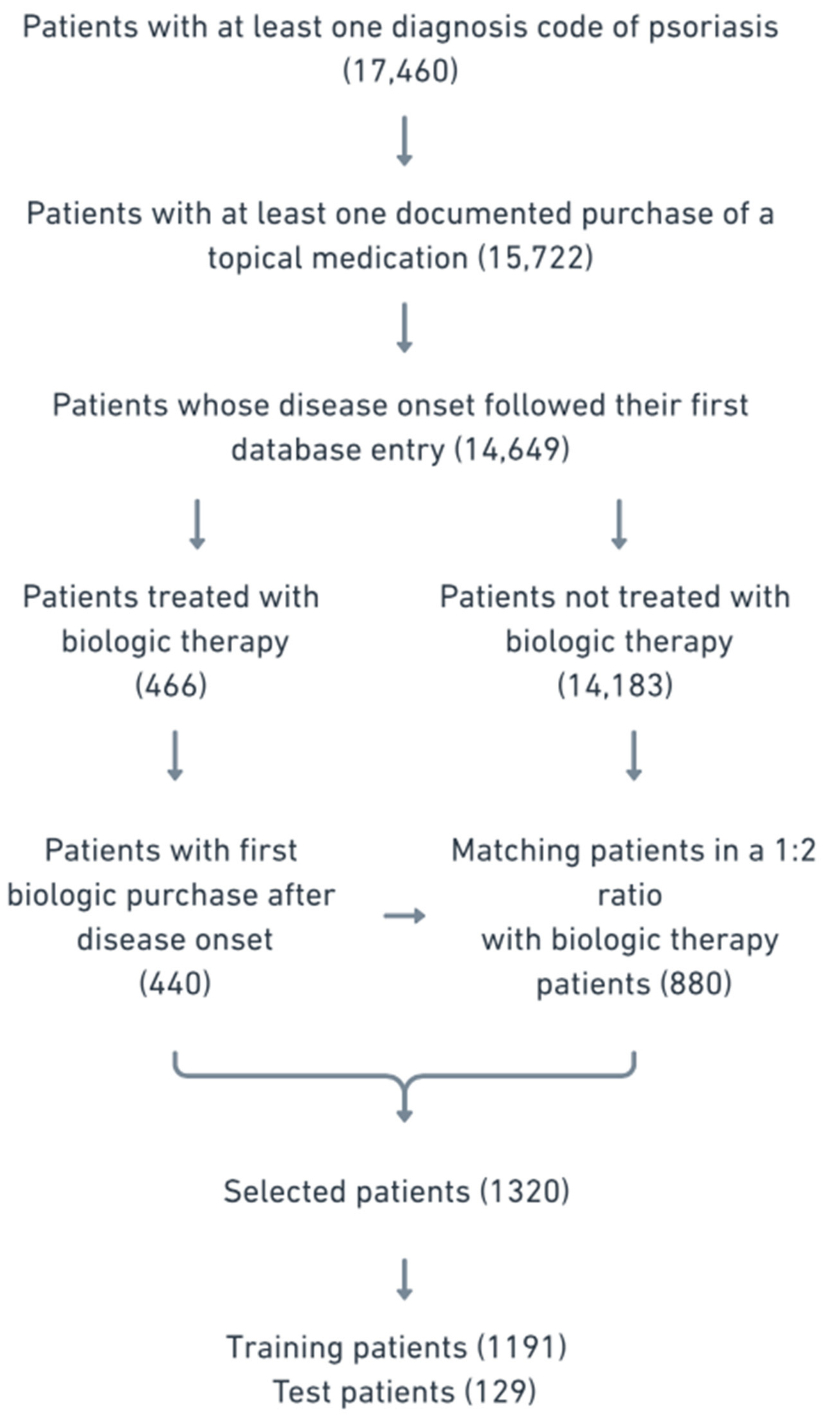

Supplementary Figure S1. Graphical Representation of the Cohort Selection Process

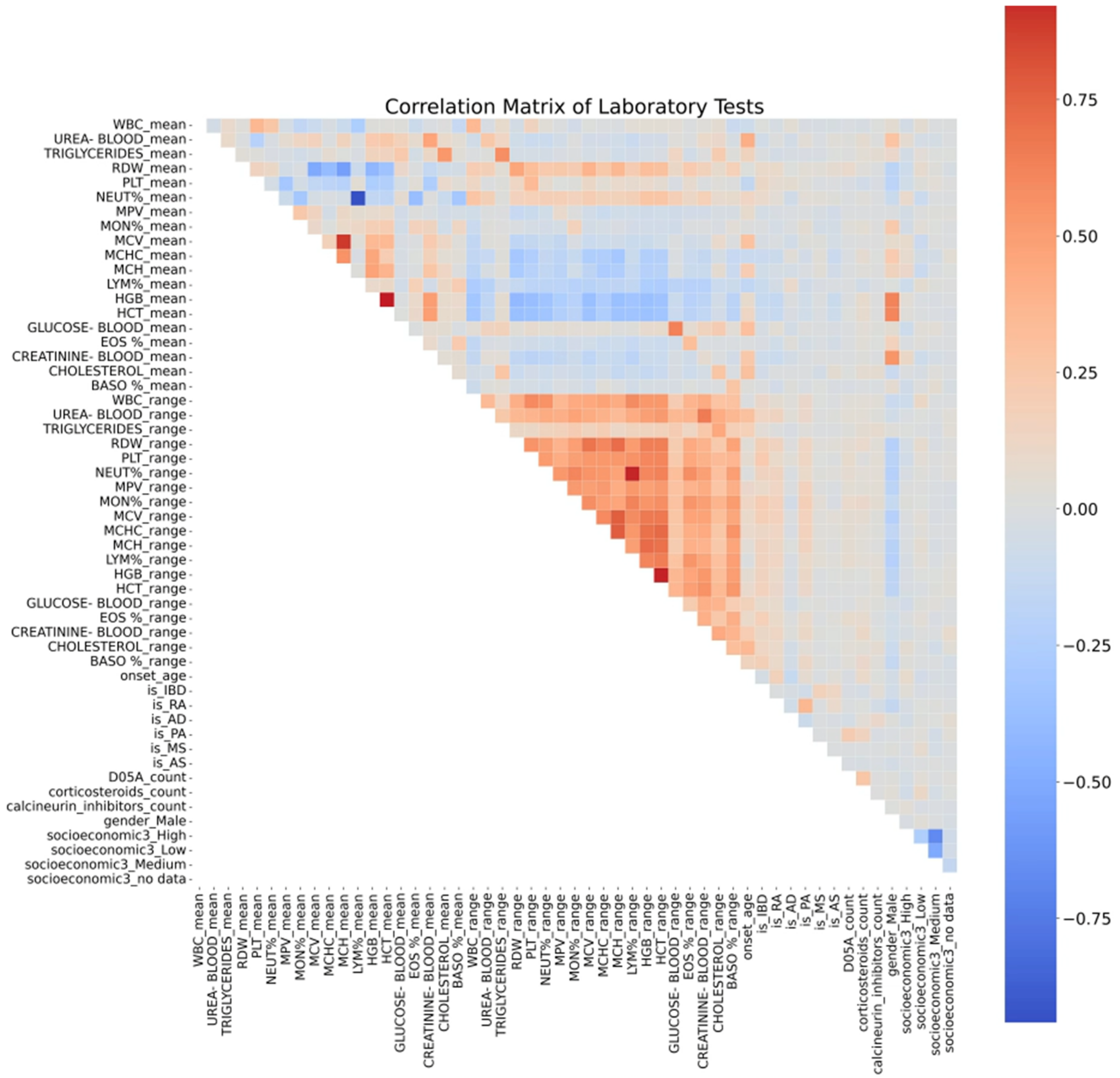

Supplementary Figure S2. Correlation Matrix of Laboratory, Demographic, and Clinical Features from the First 5 Years After Psoriasis Onset (Training Data) The heatmap shows pairwise Pearson correlation coefficients. Red indicates positive correlation, blue indicates negative correlation, and color intensity reflects the strength of the relationship. Features include laboratory test means and ranges, demographic factors, medication counts, disease indicators, and socioeconomic status.

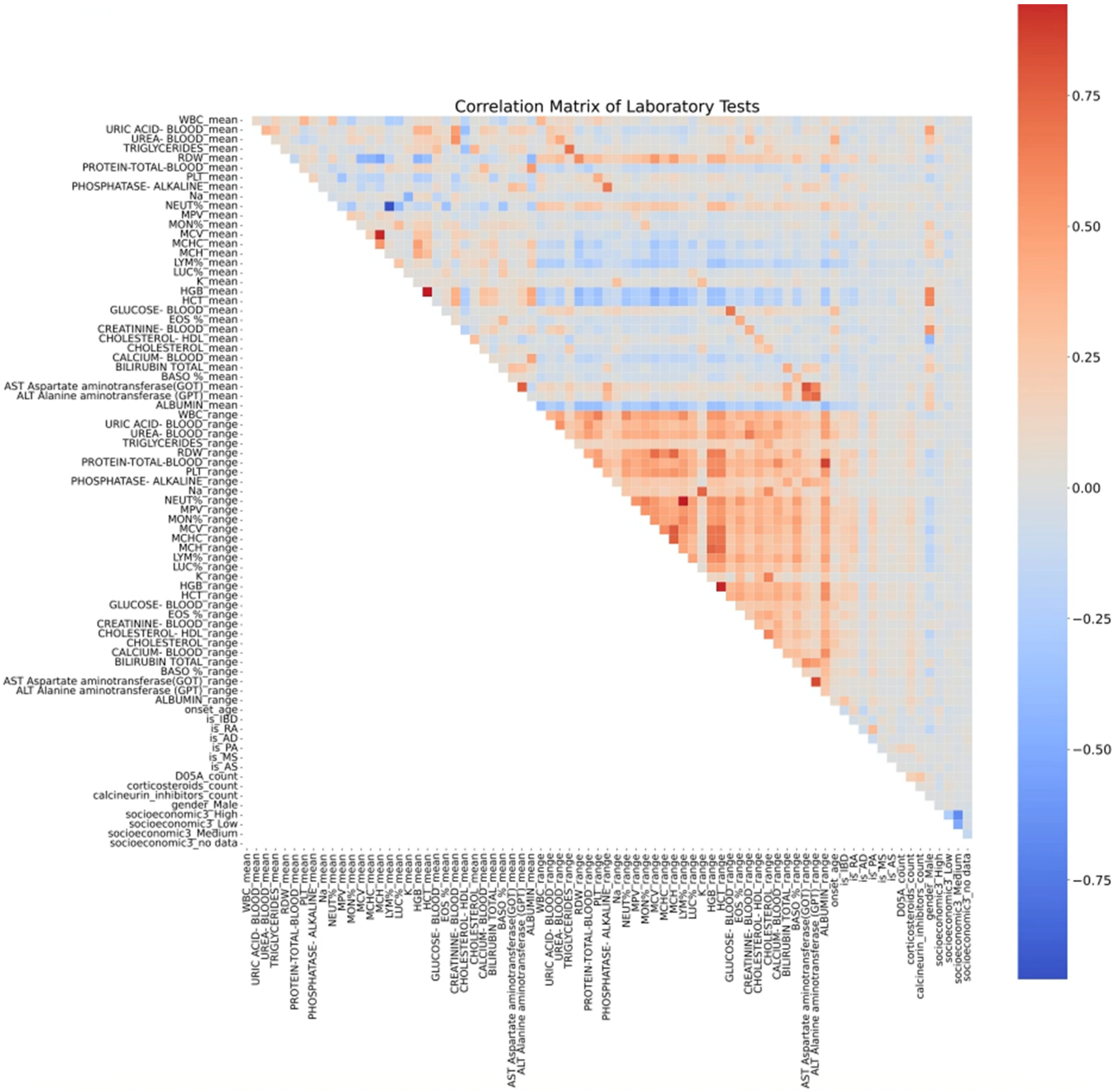

Supplementary Figure S3. Correlation Matrix of Laboratory, Demographic, and Clinical Features from the 5-Year Prior to the Index Date (Training Data)

The heatmap shows pairwise Pearson correlation coefficients. Red indicates positive correlation, blue indicates negative correlation, and color intensity reflects the strength of the relationship. Features include laboratory test means and ranges, demographic factors, medication counts, disease indicators, and socioeconomic status.

Supplementary Table S1. Features with High Intercorrelation (Pearson's |r| > 0.8) Among Training Data Recorded in the First 5 Years after Onset.

Feature 1	Feature 2	Correlation
NEUT% mean	LYM% mean*	-0.94
MCV mean	MCH mean*	0.89
HGB mean	HCT mean*	0.96
NEUT% range	LYM% range*	0.94
HGB range	HCT range*	0.95

Features marked with an asterisk (*) were excluded from the analysis to prevent multicollinearity

Supplementary Table S2. Features with High Intercorrelation (Pearson's |r| > 0.8) Among Training Data Recorded in the Interval of 5 Years Before the Index Date.

Feature 1	Feature 2	Correlation
NEUT% mean	LYM% mean*	-0.94
MCV mean	MCH mean*	0.925
HGB mean	HCT mean*	0.96
**AST Aspartate aminotransferase(GOT)** mean	AST Aspartate aminotransferase(GOT) range*	0.8
PROTEIN-TOTAL-BLOOD range*	ALBUMIN range	0.87
NEUT% range	LYM% range*	0.95
HGB range	HCT range*	0.93
AST Aspartate aminotransferase(GOT) range*	ALT Alanine aminotransferase (GPT) range	0.84

Features marked with an asterisk (*) were excluded from the analysis to prevent multicollinearity

Supplementary Table S3. Laboratory Tests Used to Build the Models in the First Stage (training data recorded in the first 5 years after onset)

Test Name (Abbreviation)	Biological Therapy Group (mean ± std. dev.)	Conventional Therapy Group (mean ± std. dev.)
**White Blood Cells (WBC)**	7.85 ± 2.14	7.53 ± 1.92
**Urea - Blood (UREA)**	29.82 ± 7.93	29.72 ± 7.42
**Triglycerides (TRIGLYCERIDES)**	149.38 ± 100.76	137.52 ± 98.9
**Red Cell Distribution Width (RDW)**	13.85 ± 1.27	13.52 ± 1.08
**Platelets (PLT)**	267.2 ± 74.76	256.26 ± 60.6
**Neutrophils % (NEUT%)**	60.04 ± 8.49	58.78 ± 0.09
**Mean Platelet Volume (MPV)**	9.13 ± 1.13	9.10 ± 1.2
**Monocytes % (MON%)**	6.38 ± 1.62	6.36 ± 1.77
**Mean Corpuscular Volume (MCV)**	85.6 ± 6.03	85.88 ± 5.66
**Mean Corpuscular Hemoglobin Concentration (MCHC)**	33.15 ± 1.10	33.42 ± 0.96
**Mean Corpuscular Hemoglobin (MCH)***	28.33±2.59	28.7±2.12

**Hemoglobin (HGB)**	13.72 ± 1.49	13.96 ± 1.49
**Glucose - Blood (GLUCOSE)**	96.22 ± 21.95	95.31 ± 21.95
**Eosinophils % (EOS%)**	3.03 ± 1.88	3.02 ± 1.72
**Creatinine - Blood (CREATININE)**	0.82 ± 0.18	0.84 ± 0.19
**Cholesterol - Total (CHOLESTEROL)**	186.02 ± 39.54	186.81 ± 37.05
**Basophils % (BASO%)**	0.53 ± 0.23	0.57 ± 0.26

* The value of Mean Corpuscular Hemoglobin (MCH) mean was excluded from the analysis to prevent multicollinearity

Supplementary Table S4. Laboratory Tests Used to Build the Models in the Second Stage (training data recorded in the interval of 5 years before the index date)

Test Name (Abbreviation)	Biological Therapy Group (mean ± std. dev.)	Conventional Therapy Group (mean ± std. dev.)
**White Blood Cells (WBC)**	7.76 ± 1.90	7.40 ± 1.86
**Uric Acid - Blood (URIC ACID)**	5.52 ± 1.43	5.39 ± 1.38
**Urea - Blood (UREA)**	30.64 ± 8.45	30.77 ± 8.57
**Triglycerides (TRIGLYCERIDES)**	141.38 ± 71.84	135.32 ± 76.23
**Red Cell Distribution Width (RDW)**	14.05 ± 1.23	13.61 ± 1.07
**Protein - Total - Blood (PROTEIN-TOTAL)***	7.21 ± 0.46	7.23 ± 0.42
**Platelets (PLT)**	263.10 ± 74.17	248.47 ± 61.10
**Alkaline Phosphatase (PHOSPHATASE-ALKALINE)**	85.84 ± 38.81	82.48 ± 39.26
**Sodium (Na)**	140.09 ± 2.65	140.12 ± 3.16
**Neutrophils % (NEUT%)**	61.00 ± 7.63	58.60 ± 8.15
**Mean Platelet Volume (MPV)**	9.33 ± 1.03	9.45 ± 1.14
**Monocytes % (MON%)**	6.54 ± 1.52	6.47 ± 1.51
**Mean Corpuscular Volume (MCV)**	86.55 ± 5.73	86.74 ± 5.66
**Mean Corpuscular Hemoglobin Concentration (MCHC)**	32.86 ± 0.96	33.13 ± 0.97
**Mean Corpuscular Hemoglobin (MCH)***	28.46±2.21	28.74±2.10
**Large Unstained Cells % (LUC%)**	1.75 ± 0.55	1.87 ± 0.60
**Potassium (K)**	4.44 ± 0.35	4.52 ± 1.58
**Hemoglobin (HGB)**	13.64 ± 1.42	13.90 ± 1.47
**Glucose - Blood (GLUCOSE)**	99.20 ± 23.46	99.92 ± 25.78
**Eosinophils % (EOS%)**	3.03 ± 2.00	3.01 ± 1.79
**Creatinine - Blood (CREATININE)**	0.81 ± 0.19	0.83 ± 0.20
**High-Density Lipoprotein Cholesterol (CHOLESTEROL-HDL)**	44.38 ± 9.89	47.42 ± 11.47
**Cholesterol - Total (CHOLESTEROL)**	179.63 ± 35.45	180.22 ± 34.21
**Calcium - Blood (CALCIUM)**	9.39 ± 0.35	9.43 ± 0.41
**Bilirubin - Total (BILIRUBIN TOTAL)**	0.59 ± 0.24	0.66 ± 0.32
**Basophils % (BASO%)**	0.52 ± 0.25	0.54 ± 0.28

**Aspartate Aminotransferase (GOT)***	23.98 ± 9.42	24.85 ± 15.82
**Alanine Aminotransferase (GPT)**	26.30 ± 15.37	25.57 ± 21.61
**Albumin (ALBUMIN)**	4.22 ± 0.32	4.32 ± 0.31

* The values of Mean Corpuscular Hemoglobin (MCH) mean, AST Aspartate aminotransferase(GOT) range, and PROTEIN-TOTAL-BLOOD range were excluded from the analysis to prevent multicollinearity
